# Bioconversion of stilbenes in genetically engineered root and cell cultures of tobacco

**DOI:** 10.1038/srep45331

**Published:** 2017-03-27

**Authors:** Diego Hidalgo, Ascensión Martínez-Márquez, Elisabeth Moyano, Roque Bru-Martínez, Purificación Corchete, Javier Palazon

**Affiliations:** 1 Laboratori de Fisiologia Vegetal, Facultat de Farmacia, Universitat de Barcelona, Av. Joan XXIII sn, 08028 Barcelona, Spain; 2 Plant Proteomics and Functional Genomics Group, Department of Agrochemistry and Biochemistry, Faculty of Science, University of Alicante, Alicante, Spain; 3 Departament de Ciències Experimentals i de la Salut, Universitat Pompeu Fabra, Barcelona, Spain; 4 Department of Plant Physiology, Campus Miguel de Unamuno, University of Salamanca, E-37007 Salamanca, Spain

## Abstract

It is currently possible to transfer a biosynthetic pathway from a plant to another organism. This system has been exploited to transfer the metabolic richness of certain plant species to other plants or even to more simple metabolic organisms such as yeast or bacteria for the production of high added value plant compounds. Another application is to bioconvert substrates into scarcer or biologically more interesting compounds, such as piceatannol and pterostilbene. These two resveratrol-derived stilbenes, which have very promising pharmacological activities, are found in plants only in small amounts. By transferring the human cytochrome P450 hydroxylase 1B1 (*Hs*CYP1B1) gene to tobacco hairy roots and cell cultures, we developed a system able to bioconvert exogenous *t*-resveratrol into piceatannol in quantities near to mg L^−1^. Similarly, after heterologous expression of resveratrol O-methyltransferase from *Vitis vinifera (Vv*ROMT) in tobacco hairy roots, the exogenous t-resveratrol was bioconverted into pterostilbene. We also observed that both bioconversions can take place in tobacco wild type hairy roots (pRiA4, without any transgene), showing that unspecific tobacco P450 hydroxylases and methyltransferases can perform the bioconversion of *t*-resveratrol to give the target compounds, albeit at a lower rate than transgenic roots.

Trans-resveratrol (*t*-R) (3,4′,5-trihydroxystilbene) is a phytoalexin available from a wide range of dietary sources, including mulberries, peanuts, grapes and wine. A plethora of biological activities have been attributed to *t*-R, including inhibiting the progression of cardiovascular, carcinogenic, and neurodegenerative diseases as well as the ageing process, as confirmed by several *in vitro* assays^1^. However, the transfer of the beneficial biological properties of *t*-R from *in vitro* to *in vivo* systems is restricted by its limited oral bioavailability and rapid metabolism^2^. Natural *t*-R analogues found in low abundance, such as the polyhydroxy-derivative trans-piceatannol (*t*-Pn) (Fig. 1), are significantly more bioavailable than *t-*R due to an additional hydroxyl group^3^, and they also have higher anti-cancer and cancer chemopreventive activity^4^,^5^. Indeed, part of the antitumoral effects of *t*-R are attributed to its conversion into *t*-Pn by specific cytochrome P450 hydroxylases, in particular CYP1B1, which over-expresses in a wide range of human tumors^6^. Scarcely distributed in nature, *t*-Pn occurs in plant species such as *Passiflora edulis, Cassia marginata* or *Rhodomyrtus tomentosa*, where its accumulation can be more than 1000-fold higher than in grape^1^. Its promising pharmacological activities have prompted a search for new sources of *t*-Pn, including biotechnological approaches.

Another more biologically active derivative of *t*-R is pterostilbene (3′,5′-dimethoxy-resveratrol, *t-*Pt), whose bioavailability is enhanced more than 4-fold by two methoxy groups in its structure (Fig. 1)^7^. This may explain why *t*-Pt has higher antiproliferative effects than *t*-R and a similar activity against human colon cancer cells at half the concentration^8^. Like other stilbenes, the biological activities of *t*-Pt are mainly due to its antioxidant, anti-inflammatory and anticancer properties^7^. Like *t*-Pn, *t*-Pt is scarcely distributed in nature, and is found in *Pterocarpus marsupium*, grapevine and blueberries, where its levels reach 520 ng/g^9^,^10^.

The biotechnological production of plant bioactive compounds based on plant cell and organ cultures constitutes a biosustainable source of scarce and structurally complex plant metabolites^11^. In this context, several attempts have been made to develop the biotechnological production of *t*-R in cell factories^12^. In general, *t*-R production in grapevine cell cultures is very low and needs to be enhanced by elicitors. Methyl jasmonate (MeJA) and methylated-β-cyclodextrin (MBCD) have been reported as strong inducers of *t*-R biosynthesis and accumulation, acting synergistically when added together to the plant cell cultures^13^,^14^.

Metabolic engineering contributes a potent set of tools for increasing plant secondary metabolite production in cell cultures. The use of strong promotors to overexpress genes involved in biosynthetic pathway bottlenecks is currently a common strategy for improving the production of target compounds in engineered biological systems^15^. In this scenario, Martinez-Marquez *et al*.^16^ recently showed that constitutive expression of resveratrol O-methyltransferase in *Vitis vinifera* led to the production of *t*-Pt, and the heterologous expression of the human cytochrome P450 hydroxylase 1B1 (*Hs*CYP1B1) increased *t*-Pn accumulation in elicited grapevine cell cultures.

Also recently, Li *et al*.^17^ described the production of *t*-R and *t*-Pt in engineered yeast after feeding the culture with phenylalanine, and Wang *et al*.^18^ reported the production of *t*-Pt from *t*-R and *p*-coumaric acid in two systems, engineered yeast and *Escherichia coli.* Thus, the stilbenoid biosynthetic pathway can be partially reproduced in these microorganisms by means of metabolic engineering tools.

Plant cell cultures have also been used to bioconvert exogenous substrates by exploiting the regioselective and stereospecific properties of plant enzymes as well as the vast potential of plants for biochemical reactions, including oxidation, reduction, hydroxylation, methylation and glycosylation^19^. Hairy root cultures obtained by genetic transformation of plant material with *Agrobacterium rhizogenes* can be an efficient alternative to plant cell suspensions for bioconversions due to their greater genetic/biochemical stability, high growth capacity in hormone-free culture media and relatively low cost. Transgenic cultures have been successfully used for the esterification, glycosylation, hydroxylation, etc. of various substrates, producing known or new compounds, some of them with improved biological activities^20^. Hairy root cultures have also proved useful for the expression of ectopic genes with the aim of bioconverting an abundant natural compound into a scarcely distributed derivative. An example is the efficient bioconversion of hyoscyamine into scopolamine in transgenic tobacco hairy roots carrying the hyoscyamine-6-hydroxylase gene from *Hyoscyamus muticus*^21^.

The aim of the present study was therefore to develop a biotechnological platform based on tobacco transgenic hairy roots and cell cultures and explore their capacity to bioconvert exogenous *t*-R into its hydroxylated derivative *t*-Pn and its methylated derivative *t*-Pt by the heterologous expression of the human cytochrome P450 hydroxylase 1B1 (*Hs*CYP1B1) or *Vitis vinifera* resveratrol O-methyltransferase (*Vv*ROMT) genes, respectively. According to the current SIGMA prices, *t*-Pn and *t*-Pt are 25- and 15-fold more expensive, respectively, than *t*-R^22^. Our results show that both types of engineered hairy roots were able to bioconvert *t*-R to produce *t*-Pn or *t*-Pt, and unexpectedly, the target compounds, together with piceid, a glucosylated derivative of t-R, were also generated by the biosynthetic machinery of tobacco wild type hairy roots (pRiA4).

## Materials and Methods

### Bacteria and plasmids

To infect the plant material, three strains of *Agrobacterium rhizogenes* A4 were used: wild type and two engineered strains carrying together with the pRiA4 the binary plant expression vector pK7WG2_CYP1B1 or pJCV52_ROMT (Fig. S1) for the *Hs*CYP1B1 or *Vv*ROMT genes, respectively. These were preceded by the constitutive Cauliflower mosaic virus 35S promoter, as described in Martinez-Marquez *et al*.^16^.

### Stable transformation of tobacco and hairy root cultures

**Figure 2:**
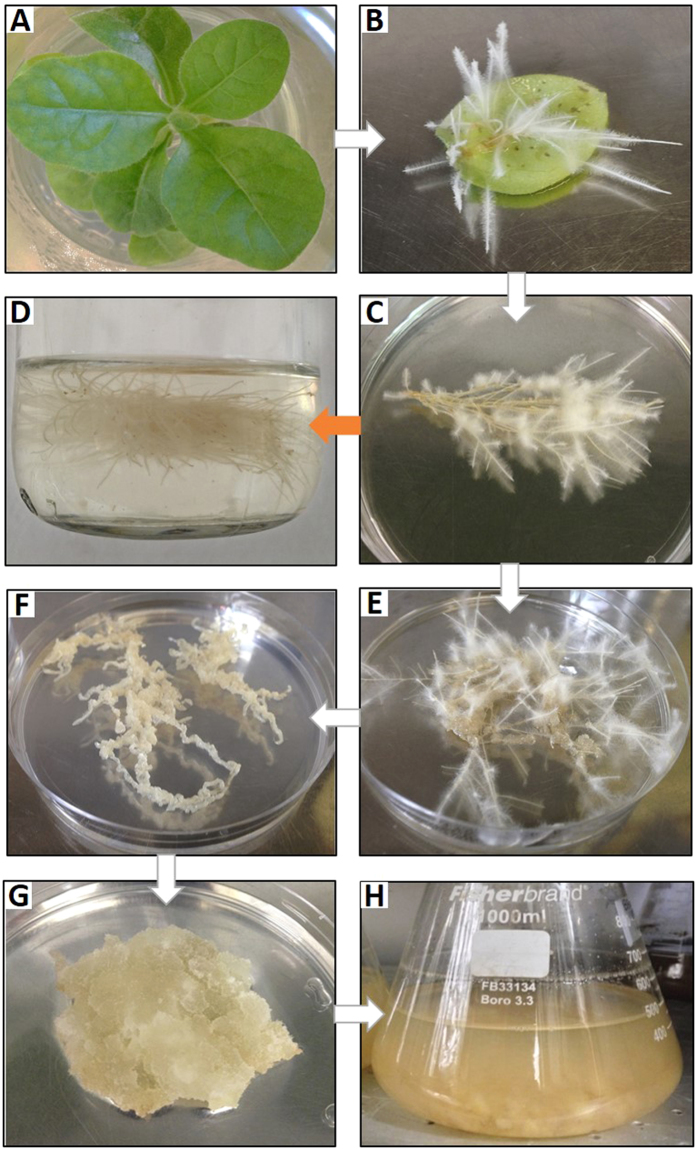
Steps for the establishment of hairy root cultures and the cell suspension derived from them. (**A**) *In vitro* plant of wild type *Nicotiana tabacum*. (**B**) Hairy roots 2–4 weeks after infection. (**C**) Hairy root line in solid MS with antibiotics. (**D**) Hairy roots in liquid MS medium, with or without MBCD. (**E**,**F**) Root dedifferentiation and callus induction in solid MS medium supplemented with NAA and KIN. (**G**) Friable callus. (**H**) Fine cell suspension in the same liquid medium, with or without MBCD.

**Figure 3:**
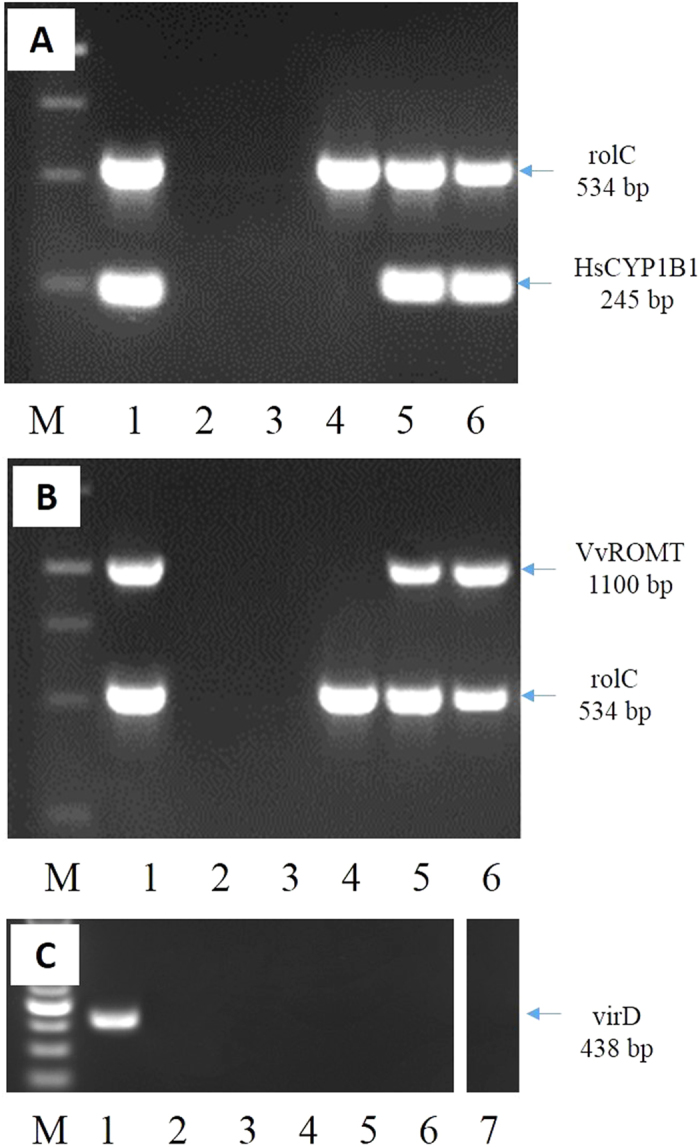
PCR analysis from genomic DNA of transgenic lines of *Nicotiana tabacum.* (**A**) (1) positive control *A. rhizogenes* (pRiA4+pK7WG2_CYP1B1), (2) negative control (DNA of root control) for the *Hs*CYP1B1 gene, (3) negative control (DNA of *Nicotiana tabacum* wild type plant) for the rolC gene, (4) hairy root control, (5–6) CYP1B1L8, CYP1B1L27 lines. (**B**) (1) positive control *A. rhizogenes* (pRiA4+pJCV52_ROMT), (2) negative control (DNA of root control) for the *Vv*ROMT gene, (3) negative control (DNA of *Nicotiana tabacum* wild type plant) for the rolC gene, (4) hairy root control, (5–6) *Vv*ROMT L3, L7 lines. (**C**) (1) positive control *A. rhizogenes*, (2) negative control (DNA of *Nicotiana tabacum* wild type plant) for the VirD gene, (3) hairy root control, (4–5) CYP1B1L8, CYP1B1L27 lines, (6–7) VvROMT L3, L7 lines.

Leaf segments of *Nicotiana tabacum* cv Xhanti plantlets grown *in vitro* on Murashige and Skoog (MS) medium^23^ were infected by direct inoculation with a needle with a wild type *A. rhizogenes* A4 strain (pRiA4), and the engineered *A. rhizogenes* (pRiA4+pK7WG2_CYP1B1) or *A.rhizogenes* (pRiA4+pJCV52_ROMT). The hairy roots began to appear after 2–4 weeks (Fig. 2). Small roots (1–2 cm) were excised and individually cultured on MS solid medium with 30 g L^−1^ of sucrose and 500 mg L^−1^ cefotaxime to eliminate the agrobacteria. After 6 rounds of subculture in MS medium supplemented with cefotaxime, the antibiotic was removed, and PCR for the virD gene was performed to confirm the elimination of *Agrobacterium* (Fig. 3C). In the case of roots obtained after infection with the recombinant *A. rhizogenes*, kanamycin (50 mg L^−1^) was used for the selection. Hairy root lines were kept in the dark at 25 °C, and after at least 6 rounds of selection by subculturing every 2 weeks in media with antibiotics, they were transferred to MS medium without antibiotics for confirmation by PCR. The growth capacity of the hairy root cultures was measured as the Growth Index (GI, harvested fresh weight/inoculum fresh weight, after 28 days of culture). Only root lines with a high GI were selected for further experiments. *A. rhizogenes* A4 carrying the empty plasmids pK7WG2 or pJCV52 was also used to obtain hairy root cultures, but as the GI and the *t*-R bioconversion of these root lines in preliminary experiments were very similar to those of the wild type *A. rhizogenes*, the latter was used for comparison with the engineered hairy root lines.

### PCR analysis

The hairy root lines were checked by PCR. The analysis was performed using the DreamTaq Green PCR Master Mix (Thermo Fisher Scientific Inc) with 1 μg DNA. Previously, genomic DNA was isolated from hairy root samples according to Dellaporta *et al*.^24^. Specific primers were used (Table S1) in the amplification of the rolC, *Hs*CYP1B1, *Vv*ROMT and virD genes. The amplification reactions were as follows: 1 cycle at 95 °C for 5 min followed by 35 cycles at 95 °C for 1 min, 57 °C for 40 sec, 72 °C for 1.30 min and an extension cycle of 10 min at 72 °C. PCR products were analyzed by electrophoresis on 1% agarose gels.

### qPCR analysis

Expression of the *Hs*CYP1B1 and *Vv*ROMT genes was verified by qPCR in the lines used in the experiments. Total RNA from plant material was isolated with TRIzol reagent (Invitrogen, Carlsbad, CA). For qRT-PCR, cDNA was prepared from 2 μg RNA treated with DNase I (Invitrogen, Carlsbad, CA) and synthesized with SuperScript III reverse transcriptase (Invitrogen, Carlsbad, CA). qRT-PCR was performed using the iTAqTM universal SYBR Green Supermix (BioRad, Hercules, CA, EEUU) in a 384-well platform system (LightCycler_ 480 Instrument; Roche), and each sample was run in triplicate, under the following conditions: 95 °C for 2 min, 40 cycles (95 °C, 10 s; 60 °C, 20 s; 72 °C, 20 s) followed by a melting curve. Gene-specific primers were designed with Primer-BLAST (Table S1). Expression levels were normalized to those of the Elongation factor 1 *α* (EF-1 *α*). Stable expression of EF-1α in the different hairy root clones and their derived cell lines was confirmed by the obtained coefficient of variation (CV) of 0.027, which is included within the CV ranking for potential internal reference genes described by Schmidt *et al*.^25^.

### Initiation and maintenance of the transgenic cell suspension

Root dedifferentiation and callus induction were performed using solid MS medium supplemented with 2.14 mg/L of naphthalene acetic acid (NAA) in combination with 0.215 mg/L of kinetin (KIN) (Fig. 2). After several subcultures, 30 g of friable callus was placed in 200 mL of liquid MS medium with the same hormones to obtain a fine cell suspension, which was subcultured every 12 days, shaken at 115 rpm and maintained at 25 °C in darkness.

### Feeding assays

In a 200 mL flask with 20 mL of liquid MS medium, with or without methylated β-cyclodextrin (MBCD) at a concentration of 5 mM (6.55 g/L), 3.4 g of roots or cells were fed with 200 μL of *t*-R sterile stock solution at 45.64 mg/mL to reach a 2 mM final concentration. Once *t*-R was added to the culture, it was shaken at 115 rpm and maintained at 25 °C in darkness up to the sampling times: 4, 8, 24, 40 and 56 h.

### Extraction and determination of stilbenoids

To extract stilbenoids from the culture medium, 1 mL of ethyl acetate was added per 4 ml of the medium, stirring vigorously, and the apolar phase was collected. The extraction was repeated once more, and the apolar phases were combined and evaporated as described in Martinez-Marquez *et al*.^16^.

The roots were frozen, freeze-dried and crushed. 50 mg of lyophilized plant material was placed in a tube with two volumes of 100% methanol, sonicated for 30 min to allow the methanol to penetrate the plant tissues, and the supernatant was collected. Again, two volumes of methanol were added and sonicated for 15 min. The methanolic extracts were pooled and evaporated. In order to measure the accuracy of the extraction method, a precisely weighed quantity of *t*-R was added to the culture medium, with or without MBCD, and extracted at different times. At time 0, just after the *t*-R addition, the *t*-R recovery was higher than 95%, demonstrating the efficiency of the extraction method employed.

For stilbenoid extraction from cells, four parts of 100% methanol were added per g of fresh weight, with stirring at 115 rpm for 24 h. The methanol extract was filtered and brought to dryness^16^. All samples were resuspended in 1 mL of 80% methanol and sonicated for 30 min and filtered through a 0.22 μm PVDF filter just before analysis.

Stilbenes were determined by a Linear Ion Trap Quadrupole LC/MS/MS Mass Spectrometer, 4000 Q TRAP of AB Sciex Instruments with MRM scan type in negative mode. Standards of t-R, *t*-Pn, *t*-Pt and piceid from LGC STANDARDS, S.L.U. were used to prepare the calibration curves described in Table S2. The gradient used in this system is described in Table 1. The mobile phases were A: H_2_O + 0.05% acetic acid and B: acetone:acetonitrile (70:30). The column was a Luna 3 μm C18 (2) 100 A 50 × 2.00 mm s/n: 008–4251-B0 with a temperature of 60 °C and injection volume of 10 uL. The transitions and retention time are described in Table S3 and Fig. S2. Stilbenoid contents are expressed as μg L^−1^ in both cells and culture medium to facilitate the calculation of the total amount of stilbenoids in the cultures.

### Statistical analysis

This was performed with Excel software. All data are the average of three measurements + SE. The multifactorial ANOVA analysis followed by the Tukey multiple comparison test were used for statistical comparisons. A p-value of <0.05 was assumed for significant differences.

## Results

### Establishment of transgenic root cultures of tobacco

**Figure 4:**
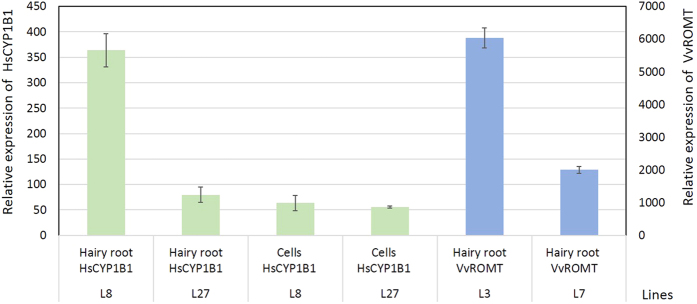
qPCR analysis of the transcript levels of the *Hs*CYP1B1 gene in transgenic hairy root lines CYP1B1L8 and CYP1B1L27 and in their respective derived cell lines. In light grey, the *Vv*ROMT gene transcript levels in transgenic hairy root lines ROMTL3 and ROMTL7. The control lines were used as a negative control to verify non-specific amplification (data not shown). Data are the mean of three independent replicates ± SE.

Tobacco hairy root cultures were established by the infection of leaf segments with *Agrobacterium rhizogenes,* harbouring the pRiA4 plasmid alone (wild type), or together with pK7WG2_CYP1B1 or pJCV52_ROMT. All the *A. rhizogenes* were able to induce hairy roots after a period of 2–4 weeks (Fig. 2). Fast-growing root lines (GI > 4, Table S4), wild type or carrying the recombinant plasmid, were selected and their transgenic nature was determined by PCR. Fig. 3 shows a band of 534 bp corresponding to rolC of *A. rhizogenes* in both wild type (pRiA4) and transgenic roots (pRiA4+pK7WG2_CYP1B1), whereas the band of 245 bp corresponding to the *Hs*CYP1B1 gene was observed only in root lines genetically transformed with the binary vector (Fig. 3A). Also, the band of 1100 bp corresponding to the *Vv*ROMT gene was only observed in the hairy root cultures infected with the corresponding agrobacteria (Fig. 3B). All the lines tested negative for the virD gene, indicating the absence of agrobacteria in the hairy root cultures (Fig. 3C). These transgenic lines, as well as some of the wild type lines, were selected for further analysis. All the obtained root lines showed the classical hairy root phenotype, a high growth capacity (Fig. 2, Table S4) and the corresponding gene expression (Fig. 4).

From these, two selected transgenic root lines carrying the *Hs*CYP1B1 gene (CYP1B1L8 and CYP1B1L27), two lines carrying the *Vv*ROMT gene (ROMTL3 and ROMTL7) and two wild type (pRiA4 alone) lines were fed with 2 mM (456.4 mg L^−1^) of *t*-R, and samples were taken at different intervals of the culture during a period of 4–56 h.

### Bioproduction of *t*-piceatannol in hairy roots and their derived cell lines

**Figure 5:**
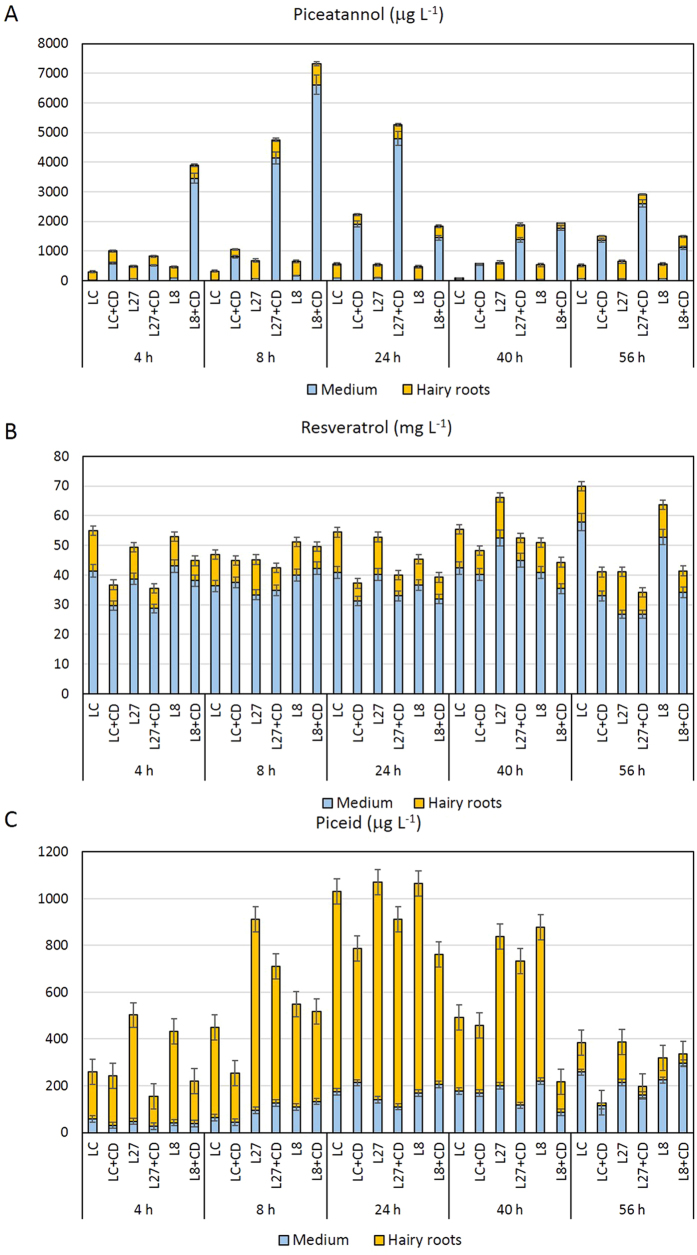
Time course of *t*-piceatannol (**A**), *t*-resveratrol (**B**) and *t*-piceid (**C**) contents in transgenic and wild type tobacco hairy root cultures. LC, A4 wild type root line. L7 and L8, CYP1B1L7 and CYP1B1L8 transgenic root lines. CD, root lines treated with MBCD. Data are the mean of 3 biological replicates ± SE.

The selected transgenic root lines heterologously expressing the *Hs*CYP1B1 gene were able to actively bioconvert the added *t*-R into *t*-Pn, especially when treated with MBCD (Fig. 5A). The highest bioconversion levels were achieved in the MBCD-supplemented CYP1B1L8 line at 8 h, when the *t*-Pn content was higher than 7 ± 0.46 mg L^−1^. At the same time, the CYP1B1L27 line reached a *t*-Pn content of 4.7 ± 0.29 mg L^−1^, which increased up to 5.2 ± 0.24 mg L^−1^ at 24 h, after which levels decreased significantly (p < 0.01). In the transgenic cultures treated with MBCD, most of the *t*-Pn was released to the culture medium, whereas the significantly lower (p < 0.01) levels of *t*-Pn produced by the untreated cultures remained mainly inside the roots (Fig. 5A). Unexpectedly, wild type hairy root cultures (without the *Hs*CYP1B1 gene) were also able to biotransform *t*-R into *t*-Pn, although at a lower rate (0.4%) than the transgenic lines CYP1B1L8 (1.6%) and CYP1B1L27 (1.4%). As before, *t*-Pn levels were also significantly higher (p < 0.05) in the MBCD-supplemented wild type cultures and accumulated mainly in the culture medium (Fig. 5A).

Regarding the fate of exogenously added resveratrol in the hairy root cultures (Fig. 5B), *t*-R taken up by the cells was partially metabolized into *t*-Pn and probably other compounds, but this stilbene was also found in the culture medium. In most cases, the remaining *t*-R contents were lower in the MBCD-treated than in the untreated cultures and accumulated mainly outside the cells. For example, the remaining *t*-R in transgenic CYP1B1L27 root cultures was 52 ± 3.26 mg L^−1^ after 24 h of treatment, 77% of which accumulated in the culture medium, whereas when the same line was treated with MBCD, the *t*-R decreased to 40 ± 2.3 mg L^−1^, 83% being found in the culture medium (Fig. 5B).

The presence of piceid, the glucoside of *t*-R, was detected in both transgenic and wild type hairy root cultures (Fig. 5C). Piceid levels peaked 24 h after the addition of the substrate and then decreased until the end of the culture period (56 h). Glucosylation of *t*-R in the cultures devoid of MBCD was higher than in MBCD-treated cultures, and it was probably a way of detoxifying the excess of exogenously added t-R. Levels of piceid were significantly lower (p < 0.05) than *t*-Pn in the transgenic cultures (pRiA4+pK7WG2_CYP1B1), whereas wild type cultures (pRiA4) showed a similar content of both *t*-R derivatives. In contrast with *t*-Pn, piceid accumulated mainly intracellularly, even in the MBCD-treated cultures (Fig. 5C). The presence of *t*-Pn in the wild type tobacco cultures, and piceid and *t*-Pt in both wild type and CYP1B1 root cultures suggests that unspecific hydroxylases and glucosidases from tobacco can transform the exogenous substrate *t*-R into these derivatives. However, the efficiency of these bioconversions was up to 24-fold lower compared with, for example, the capacity of the transgenic root CYP1B1L8 line to biotransform *t*-R into *t*-Pn. Despite the considerable variability among the different control and transgenic root lines, we can infer that the high *t*-Pt production was due to the ectopic expression of the *Hs*CYP1B1 gene, since the average yield of the transgenic CYP1B1 lines (1888 ± 427 μg L^−1^) was significantly higher (p < 0.05) than that of the control (819 ± 138 μg L^−1^). It was thus demonstrated that the transgene expression effectively increased the bioconversion of *t*-R into *t*-Pt.

**Figure 6:**
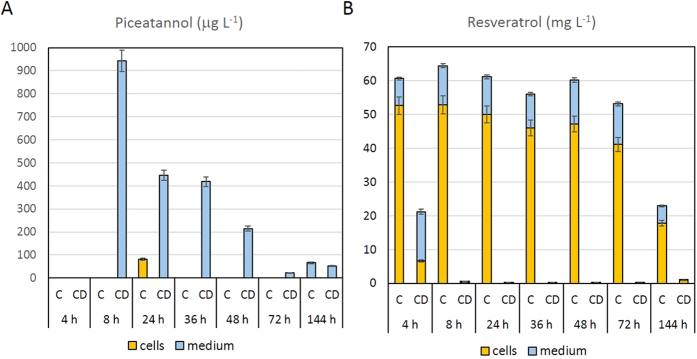
Time course of *t*-piceatannol production (**A**) and t-resveratrol accumulation (**B**) in transgenic tobacco cell cultures. (**C**) control conditions, treatment without MBCD. CD, MBCD treatment. Data are the mean of 3 biological replicates ± SE.

The most productive hairy root line (CYP1B1L8) was subjected to a hormonal treatment for dedifferentiation and callus induction. The friable calli were then disintegrated and a cell suspension line obtained (Fig. 2). Transgenic cell suspension cultures grew actively, reaching a growth rate similar to the parental hairy roots (Table S4). The cell line, with or without MBCD, was fed with the same concentration of *t*-R as the hairy root cultures to investigate its capacity to bioconvert this substrate to the hydroxylate derivative *t*-Pn. Like the CYP1B1L8 root line, its derived cell suspension was able to convert *t*-R into *t*-Pn but the production of this system was 8-fold lower than that of the original root line (Fig. 6A). In the MBCD-treated cell cultures, *t*-Pn accumulated in small quantities in the medium. Its maximum accumulation was at 8 h after feeding the culture with *t*-R, after which it decreased significantly until the end of the culture (p < 0.01). In absence of MBCD, only a low amount of *t*-Pn was detected, 24 h after the addition of the precursor (Fig. 6A). Overall, the cell suspension derived from the hairy root line L8 showed only a limited capacity to bioconvert *t*-R into *t*-Pn.

Dedifferentiation of the roots to obtain the cell suspension also affected the exogenous *t*-R accumulation pattern (Fig. 6B). In contrast with the hairy roots, the derived cell suspension culture accumulated *t*-R mainly inside the cells. When treated with MBCD, a small amount of *t*-R remained in the culture medium 4 h after feeding, and only traces were detected inside the cells (Fig. 6B). These results suggest a very low stability of *t*-R outside the cells, and most of the *t*-R remaining in the culture medium had probably degraded 4 h after the feeding treatment. No piceid was detected in any cell or media samples of the *t*-R-fed cultures, showing that the derived cell suspension lost the capacity to glycosylate the *t*-R, in contrast with the hairy root cultures.

### Bioproduction of *t*-pterostilbene in hairy root cultures

**Figure 7:**
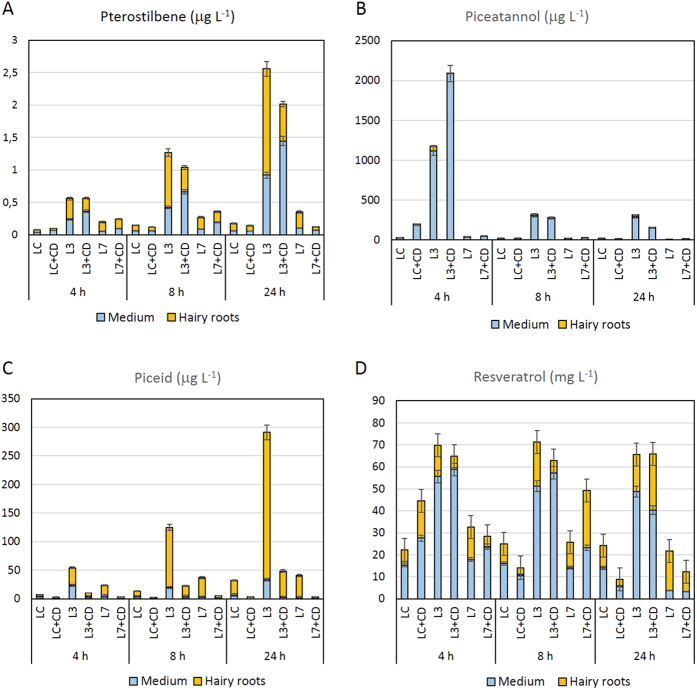
Time course of *t-*pterostilbene (**A**), *t*-piceatannol (**B**), *t*-resveratrol (**C**) and *t*-piceid (**D**) contents in transgenic and wild type tobacco hairy root cultures. LC, A4 wild type root line. L7 and L8, ROMTL7 and ROMTL8 transgenic root lines. CD, root lines treated with MBCD. Data are the mean of 3 biological replicates ± SE.

Heterologous expression of the *Vv*ROMT gene in hairy root cultures fed with *t*-R led to the bioconversion of this stilbene to its methoxylated derivative *t*-Pt, which was found both inside the roots and in the culture medium, with a maximum production reached by the transgenic root line L3, 24 h after *t*-R feeding (Fig. 7A). In this experiment, the incubation period was not extended because we had previously observed that after 24 h the newly produced stilbene contents in the cultures decreased (data not shown). As well as the control and *Hs*CYP1B1 hairy roots (Fig. 5A), the cultures carrying the transgene *Vv*ROMT were also able to synthesize *t*-Pn and piceid in even greater quantities than *t*-Pt. In particular, root line L3 reached a *t*-Pn content of 2 ± 0.14 mg L^−1^ (Fig.7B), which was very similar to that of the control root line described in the previous experiment (Fig. 5A). The same line *Vv*ROMTL3 also showed the highest *t*-Pt production (Fig. 7A). In this experiment, MBCD significantly increased (p < 0.01) the *t*-Pt content in the culture medium, generally without increasing the total yield of the target compounds in the cultures (Fig. 7A). In hairy roots carrying the *Vv*ROMT gene, as in the case of the *Hs*CYP1B1 gene, partial *t*-R degradation occurred, although *t*-R quantities of up to 70 ± 2.16 mg L^−1^ remained in the culture 24 h after feeding (Fig. 7D).

## Discussion

Tobacco is a model plant system easily transformed by *A. rhizogenes* to produce hairy root cultures. This trait may be harnessed for the heterologous expression of foreign genes harbored in engineered *A. rhizogenes*^26^. The derived genetically transformed cultures exhibit a high growth capacity and genetic stability for long periods^27^,^28^. The tobacco hairy root cultures we engineered to ectopically express the human CYP1B1 gene had the capacity to bioconvert *t*-R into *t*-Pn with a yield of up to 7 ± 0.46 mg L^−1^, and others expressing the ROMT gene from *V. vinifera* were able to biosynthesize *t*-Pt, reaching a content of 2.6 ± .019 μg L^−1^. Although low, the concentrations of *t*-Pt were more than 25-fold higher than those achieved in the control hairy roots. This biotechnological system thus proved to be suitable for the production of *t*-Pn and, on a lower scale, *t*-Pt, both compounds with promising biological activities and scarcely distributed in nature^1^.

Recently, in a similar approach, Martinez-Marquez *et al*.^16^ reported a 200-fold enhancement of *t*-Pn production in grapevine cell cultures by heterologous expression of the *Hs*CYP1B1 gene, and the presence of *t*-Pt in transgenic cell lines overexpressing the *Vv*ROMT gene, when both cultures were elicited with MeJA and MBCD. Although the *t*-Pn production achieved in the transgenic *V. vinifera* cell line was higher (up to 20 mg L^−1^) than in our study, the greater *t*-Pt production of our transgenic hairy roots, as well as the inherent genetic stability of the system^20^,^28^, confirm that this bioconversion process is also suitable for the production of *t*-R derivatives.

Although examples are few, hairy roots have been previously used for the bioconversion of exogenous substrates. Through the heterologous expression of the hyoscyamyne-6β-hydroxylase gene from *H. muticus*, Häkkinen *et al*.^27^ obtained the alkaloid scopolamine after feeding tobacco hairy root cultures with its precursor hyoscyamine. Similarly, hairy root cultures of *Peganum harmala* expressing tryptophan decarboxylase of *C. roseus* produced high levels of serotonin^29^, and *Beta vulgaris* hairy roots expressing the *p*-hydroxycinnamoyl-CoA hydratase/lyase (HCHL) gene from *Pseudomonas fluorescens* produced vanillin when the cultures were fed with ferulic acid^30^. Thus, our results further confirm the capacity of engineered hairy root cultures to biotransform exogenous substrates to target products with interesting biological activities.

In this work, the enhancing effects of MBCD on the bioconversion of *t*-R into *t*-Pn in tobacco hairy root cultures were also demonstrated (Fig. 5A). MBCD can act as a precursor solubilizer in biotransformation processes. For example, cell cultures of *Mucuna pruriens* bioconverted 17β-estradiol into 4-hydroxyestradiol when solubilized in β-MBCD, and *Podophyllum hexandrum* cell cultures converted a coniferyl alcohol MBCD complex into podophyllotoxin^31^. In our experiment, considering the poor solubility of *t*-R in water, the solubilizing effects of MBCD may have contributed to the improved efficiency of the hairy root cultures in biotransforming *t*-R to *t*-Pn. Other factors are also likely to have been involved, especially since MBCD did not clearly show any positive effects on *t*-Pt production.

MBCD has also been used as a permeabilizing agent acting on plant cell membranes, thus increasing the release of plant secondary metabolites such as taxol in *Taxus* spp. cell cultures^32^. This effect could be responsible for the higher extracellular *t*-Pn accumulation in the MBCD-treated cultures compared with the untreated control. MBCD may also facilitate the movement of substrates and products through cell membranes during the biotransformation processes and improve the uptake of *t*-R by the hairy roots, thus facilitating the metabolism of this compound inside the cells and its conversion to other stilbenoids like *t*-Pn. However, the positive effect of MBCD on the release of reaction products to the culture medium could negatively affect the production of the system if they are less stable in the medium. This may be the case with *t*-Pt or piceid, which were absent in grapevine cell cultures even after elicitation with MeJA and MBCD^16^.

Wild type hairy root cultures have been widely used to biotransform a range of exogenous substrates for the production of pharmaceutical ingredients, including products with enhanced solubility after hydroxylation and glycosydation^20^. In this context, the presence of small quantities of *t*-Pn and *t*-Pt, even in the control cultures, suggests that non-specific tobacco enzymes can also biotransform the supplied *t*-R into its derivatives in the absence of the corresponding transgene. Similarly, the presence of piceid (a *t*-R glucoside) in both transgenic and wild type hairy root cultures confirms the capacity of the tobacco hairy roots to glycosylate *t*-R.

Globally considered, our results show that in tobacco hairy root cultures, *t*-R and its derivatives are not metabolic end-products and may be transformed by unspecific tobacco enzymes into other known or new products. This could explain the lower *t*-Pt contents of our cultures compared with those of other biotechnological platforms based on engineered microorganisms. When the VvROMT gene was expressed in transgenic yeast and *E. coli*^18^, concentrations of 170 mg L^−1^ and 150 mg L^−1^ of pterostilbene, respectively, were reached when the cultures were fed with resveratrol^18^. Recently, the *t*-Pt biosynthetic pathway from phenylalanine was transferred to yeast, which required a dozen genetic modifications, and the engineered cultures produced up to 34 mg L^−1^ of pterostilbene^17^. However, only the non-bioactive *t*-R derivative pinostilbene^17^ was detected in these cultures and not the high added value *t*-Pt. These results suggest that bioconversion in metabolically engineered microorganisms can yield a high amount of a target compound, but the metabolic complexity of plant organisms can provide a wider range of compounds, probably including new products.

As mentioned before, plant cell cultures are widely employed for the bioconversion of naturally abundant substrates to scarcer secondary metabolites with important biological activities^19^,^33^. According to our results, the tobacco transgenic cell cultures carrying the *Hs*CYP1B1 transgene were considerably less able to biotransform *t-*R into *t*-Pn than the parental transgenic roots. Nevertheless, the low levels of *t*-R remaining in the cell cultures, especially when MBCD was added, compared with the root cultures, suggests the transgenic cells have a high capacity to metabolize *t*-R. A greater capacity to bioconvert hyoscyamine to scopolamine in hairy roots compared with the corresponding derived cell lines was also found in tobacco transgenic cultures heterologously expressing the hyoscyamine-β-hydroxylase gene from *Hyoscyamus muticus*^21^. In contrast, when comparing tobacco hairy roots and cell cultures expressing the geraniol synthase gene of *Valeriana officinalis*, Vasilev *et al*.^26^ obtained higher levels of geraniol in the cell cultures. However, in this case, the substrate (geranylgeranyl diphosphate) was generated by the plant cells and not added exogenously to the culture.

## Conclusions

Taken as a whole, our results show the possibility of developing a *t*-Pn-producing biotechnological platform based on metabolically engineered tobacco hairy roots heterologously expressing the *Hs*CYP1B1 gene, with MBCD playing an important role as a solubilizing/permeabilizing agent. The *t*-Pt production achieved was low, but this is an extremely scarce compound, even in its richest natural sources, such as blueberries, which only accumulate ng/g^7^. Thus, the developed system, based on the heterologous expression of the *Vv*ROMT gene, has potential as a biotechnological source of *t*-Pt after an optimization process. Finally, both untransformed systems were also able to biosynthesize *t*-Pn, *t*-Pt and piceid using the natural genetic capacity of the host plant to perform non-specific hydroxylations, methoxylations and glycosylations, thus demonstrating the immense capacity of plant cells to carry out biotransformations and generate known or even new products.

As previously mentioned, metabolically engineered yeast and *E. coli* cultures have been developed for *t*-R production from simple and abundant precursors such as phenylalanine and *p*-coumaric acid. However, production in these systems requires the introduction of the whole gene set of the metabolic pathway for stilbenoid synthesis. In contrast, since the direct natural *t*-R precursors, malonyl CoA and *p*-coumaryl CoA, are already found in plant tissues, heterologous production in biotechnological platforms of plant origin has the advantage of requiring the introduction of only one or two genes.

Therefore, and according with our results, it is conceivable that in the near future new biotechnological systems based on plant cell or hairy root cultures will be designed to produce *t*-R by heterologous expression of the stilbene synthase gene, as well as the resveratrol derivatives *t*-Pn and *t*-Pt, if they also carry the transgenes VvROMT and/or HsCYP1B1. In support of this hypothesis, Xiao *et al*.^34^ dramatically activated rosmarinic acid biosynthesis by the genetic manipulation of only two genes of the metabolic pathway in hairy root cultures of *Salvia miltiorrhiza*.

## Additional Information

**How to cite this article**: Hidalgo, D. *et al*. Bioconversion of stilbenes in genetically engineered root and cell cultures of tobacco. *Sci. Rep.* **7**, 45331; doi: 10.1038/srep45331 (2017).

**Publisher's note:** Springer Nature remains neutral with regard to jurisdictional claims in published maps and institutional affiliations.

## Supplementary Material

Supplementary Material

## Figures and Tables

**Figure 1:**
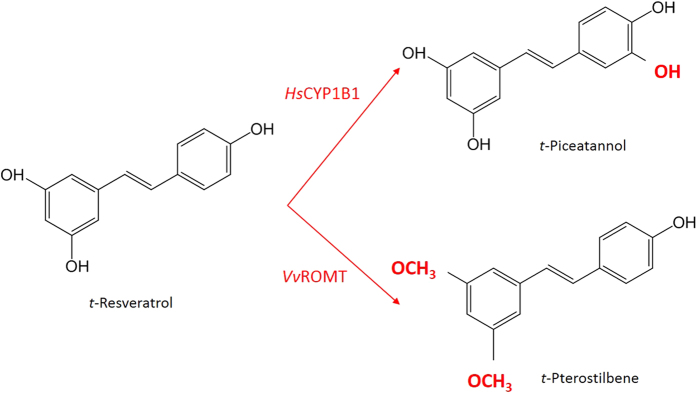
Bioconversion of t-resveratrol into *t*-piceatannol or *t*-pterostilbene by the action of the enzymes encoded by the genes *Hs*CYP1B1 and *Vv*ROMT, respectively.

**Table 1:** Gradient solvents used for analysis of stilbenes were A: H_2_O + 0.05% acetic acid; B: acetone:acetonitrile (70:30).

Step	Total Time (min)	Flow Rate (μL/min)	A (%)	B (%)
0	0	800	100	0
1	1	800	100	0
2	1.5	800	60	40
3	2.5	800	0	100
4	4.5	800	0	100
5	4.6	700	100	0
6	8	700	100	0
