# Supplementary material for: Bioconversion of stilbenes in genetically engineered root and cell cultures of tobacco
Source: Sci Rep. 2017 Mar 27;7:45331. doi: 10.1038/srep45331 (PMC5366909; doi:10.1038/srep45331)
Supplement: Supplementary Material [file srep45331-s1.pdf]

# Supplementary material:

## Bioconversion of stilbenes in genetically engineered root and cell cultures of tobacco

Diego Hidalgo, Ascensión Martínez-Márquez, Elisabeth Moyano, Roque Bru-Martínez, Purificación Corchete, Javier Palazon.

**Table S1.** Sequences of the primers used to amplify the genes by PCR and qPCR.

| Gene | Primer Sequence | Amplicon size bp | Reference |
|---|---|---|---|
| rolC | Sense<br>5'-TAACATGGCTAGAAGACGACC-3'<br>Reverse<br>5'-AAACTTGCACTCGCCATGCC-3' | 534 | 1 |
| *Hs*CYP1b1 | Sense<br>5´-CCTATGTCCTGGCCTTCCTT-3´<br>Reverse<br>5´-ACTCTGCTGGTCAGGTCCTT-3´ | 245 | 2 |
| *Vv*ROMT | Sense<br>5´- CACCATGGATTTGGCAACG-3´<br>Reverse<br>5´-AGGATAAACCTCAATGAGGGA C-3´ | 1100 | 2 |
| virD | Sense<br>5'-ATGTCGCAAGGCAGTAAGCCCA-3'<br>Reverse<br>5'-GGAGTCTTTCAGCATGGAGCAA-3' | 438 | 3 |
| Elongation factor | Sense<br>5´- TGGTCAGGAGATTGCGAAAGA GC -3´<br>Reverse<br>5´- ACGCAAAACGCTCCAATGGTG -3´ | 130 | This work |
| *Hs*CYP1b1 | Sense<br>5´- TTTTCAGTGGGCAAAAGGCG -3´<br>Reverse<br>5´- GGTGAGCCAGGATGGAGATG -3´ | 82 | This work |
| *Vv*ROMT | Sense<br>5´- CGATGATGATTTTCGCCCCG -3´<br>Reverse<br>5´- CTCCCATTCGTTCTCGTCCC -3´ | 50 | This work |

**Table S2**. Calibration curve

| Sample Name | Analyte Peak Name | Analyte Peak Area (counts) | Analyte Concentration (ng/mL) | Calculated Concentration (ng/mL) | Equation of curve |
|---|---|---|---|---|---|
| | | x | | y | |
| ppb0.39 | *t*-Resveratrol | 763.713396 | 0.39 | 0.256859 | y = 0.0009x – 0.3985<br>$R^2$ = 1 |
| ppb0.78 | | 1439.95107 | 0.78 | 0.837166 | |
| ppb1.56 | | 2121.44183 | 1.56 | 1.42198 | |
| ppb3.125 | | 3803.43042 | 3.125 | 2.86536 | |
| ppb6.25 | | 8266.67191 | 6.25 | 6.69545 | |
| ppb12.5 | | 15317.5911 | 12.5 | 12.7461 | |
| ppb25 | | 29208.628 | 25 | 24.6666 | |
| ppb50 | | 58917.2495 | 50 | 50.1608 | |
| ppb100 | | 116942.662 | 100 | 99.9547 | |
| | | x | | y | |
| ppb0.39 | Piceid | 1584.22714 | 0.39 | 0.85061 | y = 0.0003x + 0.3476<br>$R^2$ = 1 |
| ppb0.78 | | 2992.22286 | 0.78 | 1.2976 | |
| ppb1.56 | | 4941.48679 | 1.56 | 1.91642 | |
| ppb3.125 | | 9168.62143 | 3.125 | 3.25839 | |
| ppb6.25 | | 19831.2368 | 6.25 | 6.6434 | |
| ppb12.5 | | 37930.255 | 12.5 | 12.3892 | |
| ppb25 | | 76705.5246 | 25 | 24.699 | |
| ppb50 | | 146961.793 | 50 | 47.0029 | |
| ppb100 | | 318774.849 | 100 | 101.548 | |
| | | x | | y | |
| ppb0.39 | *t*-Piceatannol | 334.648096 | 0.39 | 0.756154 | y = 0.0014x + 0.3029<br>$R^2$ = 1 |
| ppb0.78 | | 655.427093 | 0.78 | 1.19057 | |
| ppb1.56 | | 1204.09439 | 1.56 | 1.93361 | |
| ppb3.125 | | 1946.55837 | 3.125 | 2.93911 | |
| ppb6.25 | | 3581.88342 | 6.25 | 5.15377 | |
| ppb12.5 | | 8616.25 | 12.5 | 11.9716 | |
| ppb25 | | 17041.7668 | 25 | 23.382 | |
| ppb50 | | 39271.8422 | 50 | 53.4874 | |
| ppb100 | | 72724.1228 | 100 | 98.7907 | |
| | | x | | y | |
| ppb0.39 | *t*-Pterostilbene | 278.803271 | 0.39 | 0.650916 | y = 0.0011x + 0.3497<br>$R^2$ = 1 |
| ppb0.78 | | 1040.26607 | 0.78 | 1.47371 | |
| ppb1.56 | | 1566.57036 | 1.56 | 2.0424 | |
| ppb3.125 | | 3292.82357 | 3.125 | 3.90769 | |
| ppb6.25 | | 5130.94016 | 6.25 | 5.89385 | |
| ppb12.5 | | 10928.5934 | 12.5 | 12.1585 | |
| ppb25 | | 23437.2971 | 25 | 25.6746 | |
| ppb50 | | 42146.6475 | 50 | 45.8909 | |
| ppb100 | | 93992.4229 | 100 | 101.912 | |

**Table S3.** Transition and retention time of stilbenes

| Stilbene | Q1 Mass (Da) | Q3 Mass (Da) | Retention time (min) |
|---|---|---|---|
| t-Resveratrol | 227.100 | 184.700 | 3.10 |
| Piceid | 389.200 | 227.100 | 2.94 |
| t-Piceatannol | 243.000 | 200.700 | 3.03 |
| t-Pterostilbene | 255.100 | 239.800 | 3.37 |

**Table S4.** Growth Index (GI) of hairy root and cell cultures after a culture period of 28 days for roots and 12 days for cells. The results are the average of 3 biological replicates±SD.

| Line | GI of hairy root after 28 days | GI of cells suspension after 12 days |
|---|---|---|
| Control 1 | 5,3 ± 0,21 | 10,1 ± 0,38 |
| Control 2 | 5,1 ± 0,20 | 9,8 ± 0,33 |
| CYP1B1 L8 | 4,7 ± 0,23 | 10,8 ± 0,35 |
| CYP1B1 L27 | 5,2 ± 0,19 | 9,8 ± 0,32 |
| VvROMT L3 | 4,8 ± 0,21 | 10,0 ± 0,30 |
| VvROMT L7 | 5,0 ± 0,20 | 9,7 ± 0,37 |

**Fig. S1**

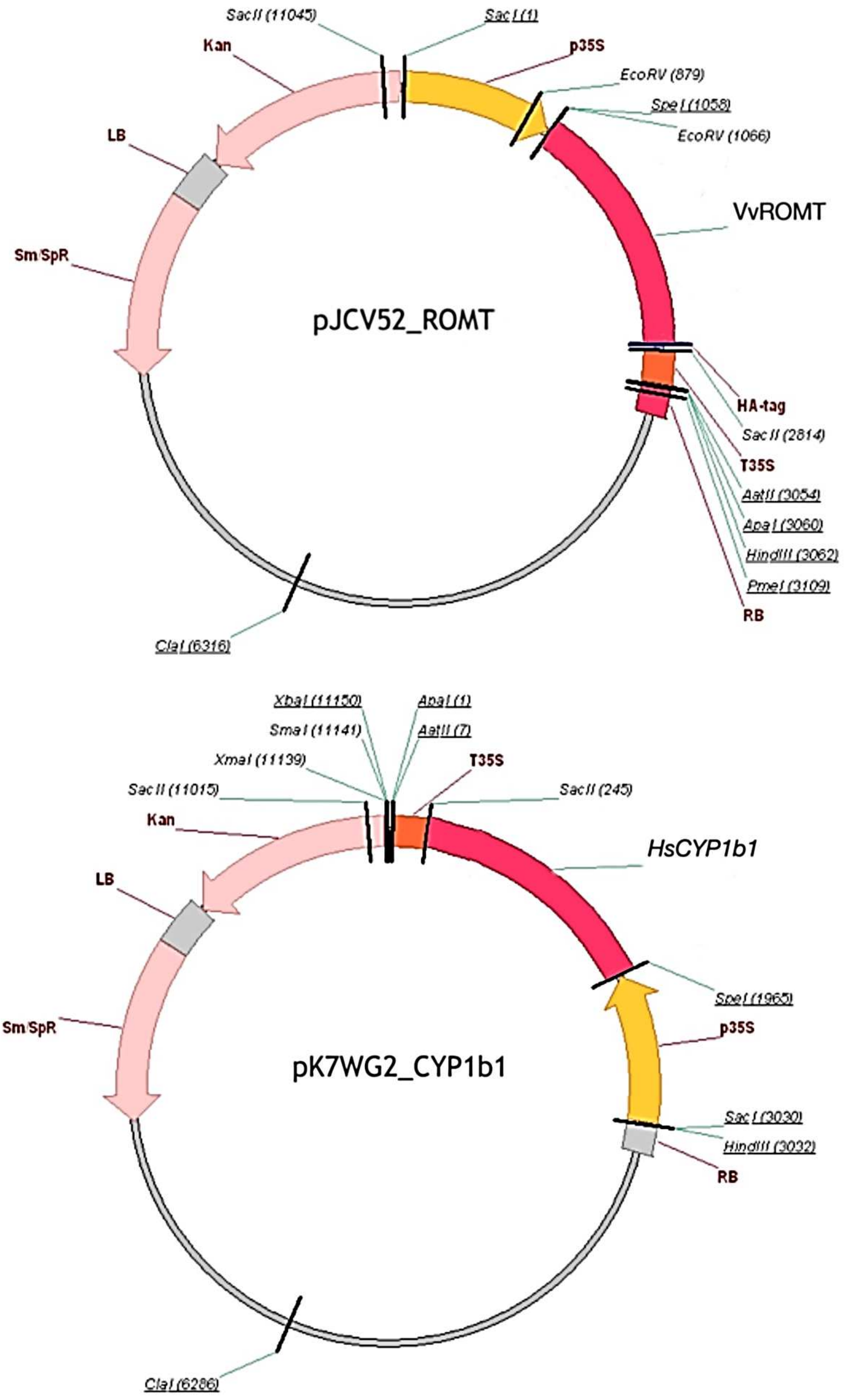


**Fig. S1:** Plant expression vector pK7WG2_CYP1B1 or pJCV52_ROMT [4].

**Fig. S2**

**A**

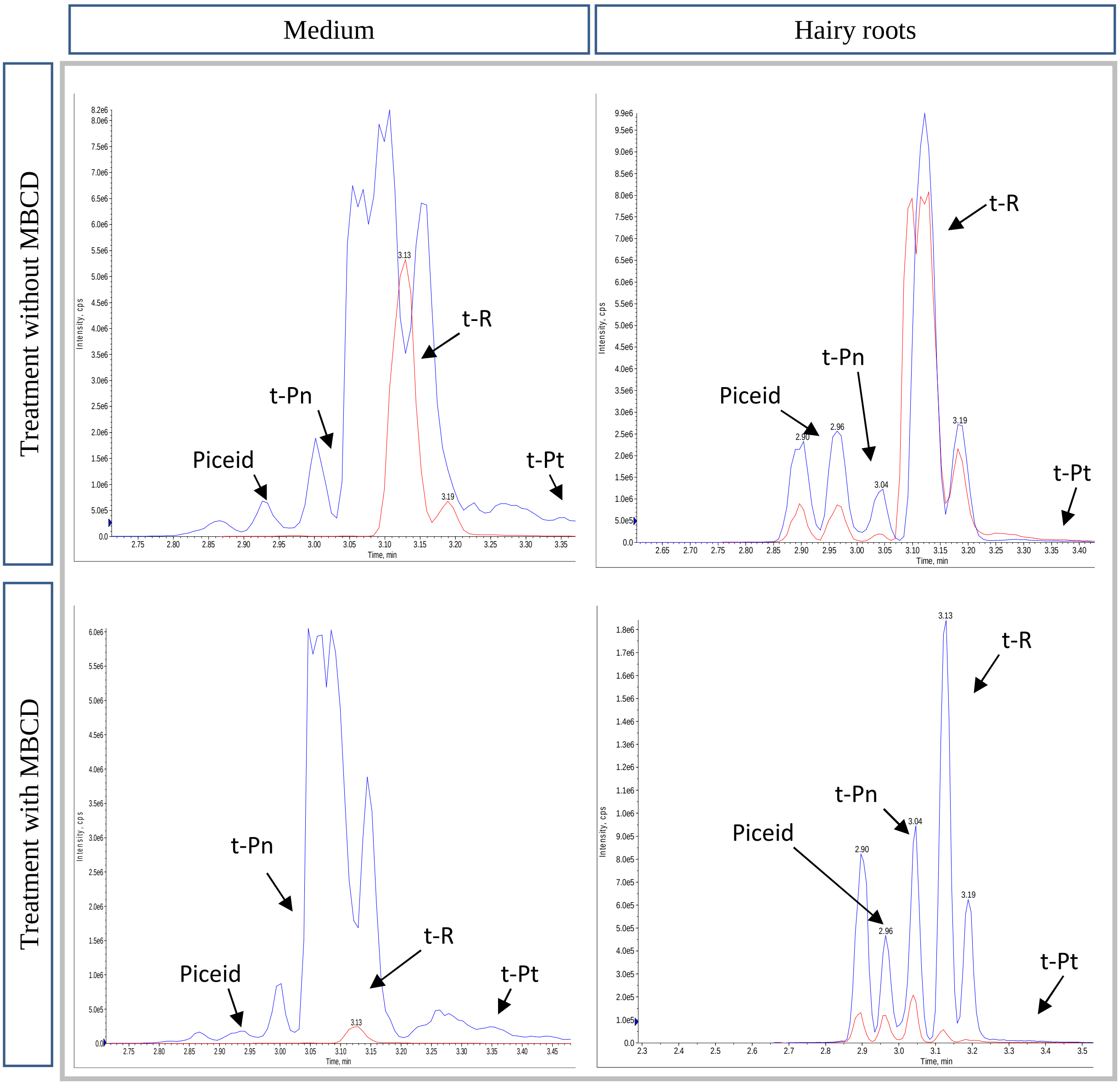
Medium
Hairy roots
Treatment without MBCD
Treatment with MBCD
Piceid
t-Pn
t-R
t-Pt
Intensity, cps
Time, min
3.13
3.19
2.90
2.96
3.04

**B**

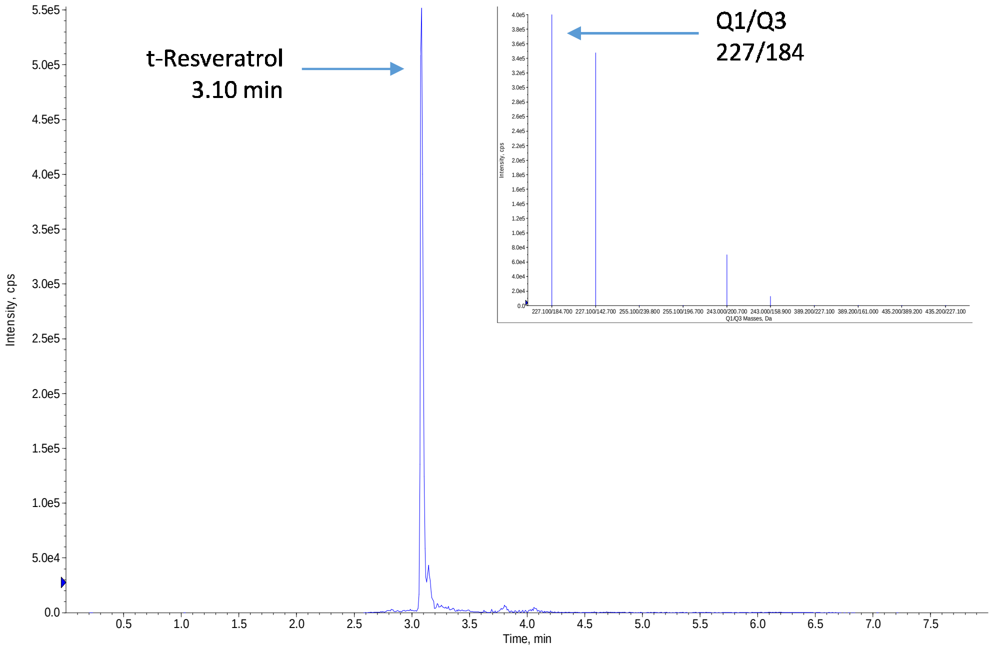
t-Resveratrol
3.10 min
Q1/Q3
227/184
Intensity, cps
Time, min
5.5e5
5.0e5
4.5e5
4.0e5
3.5e5
3.0e5
2.5e5
2.0e5
1.5e5
1.0e5
5.0e4
0.0
0.5
1.0
1.5
2.0
2.5
3.0
3.5
4.0
4.5
5.0
5.5
6.0
6.5
7.0
7.5

**C**

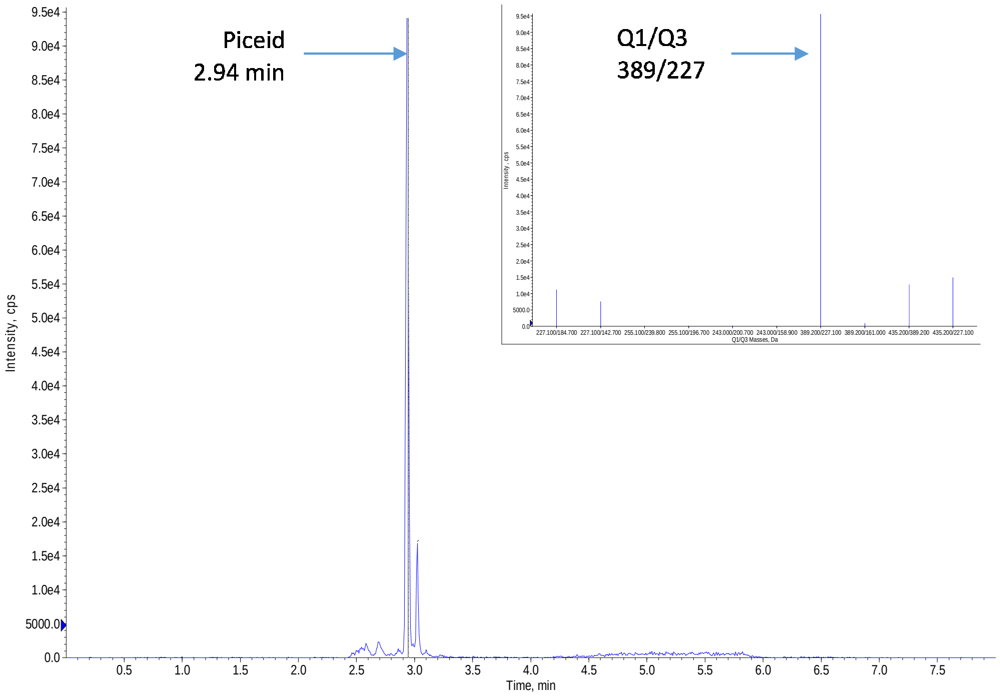
Piceid
2.94 min
Q1/Q3
389/227
Intensity, cps
Time, min
9.5e4
9.0e4
8.5e4
8.0e4
7.5e4
7.0e4
6.5e4
6.0e4
5.5e4
5.0e4
4.5e4
4.0e4
3.5e4
3.0e4
2.5e4
2.0e4
1.5e4
1.0e4
5000.0
0.0
0.5
1.0
1.5
2.0
2.5
3.0
3.5
4.0
4.5
5.0
5.5
6.0
6.5
7.0
7.5

**D**

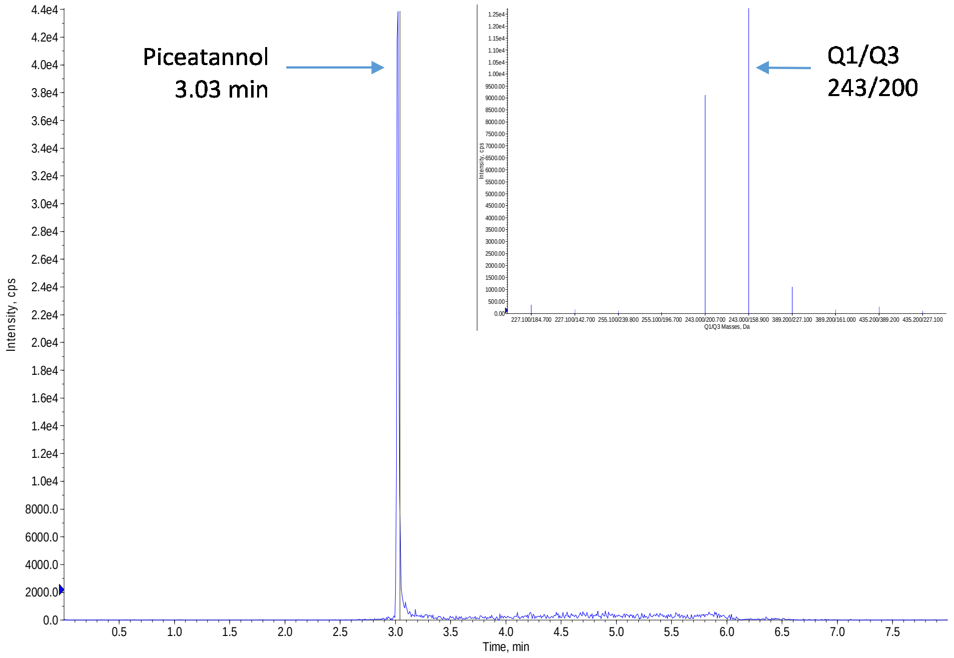


**E**

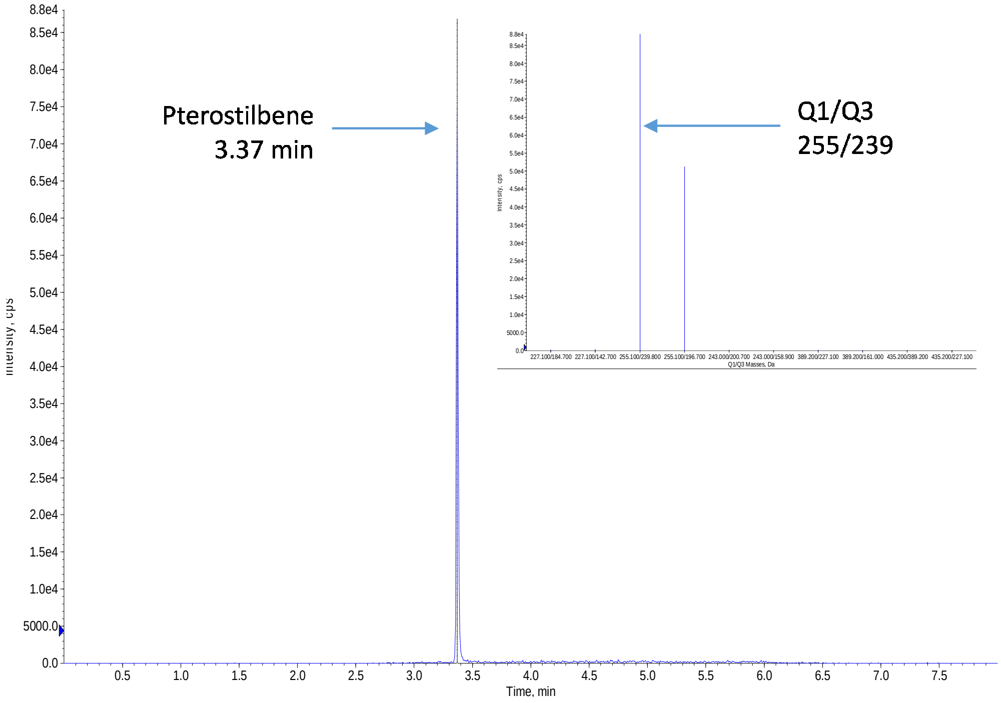


**Fig. S2. (A).**Total ion current chromatogram (TIC) of MRM from the most representative samples after 24 h of feeding assays, in red the control line and in blue the transgenic line. Extract ion chromatogram (XIC) and fragmentation pattern, (B) t-Resveratrol (Q1/Q3 227/184), (C) Piceid (Q1/Q3 389/227), (D) Piceatannol (Q1/Q3 243/200) and (E) Pterostilbene (Q1/Q3 255/239).

**Unprocessed original scans for all of the blots in figure 3.**

**Fig. 3A**

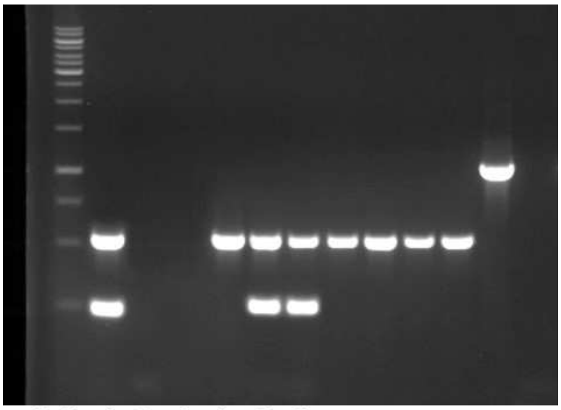

M 1 2 3 4 5 6

**Fig. 3B**

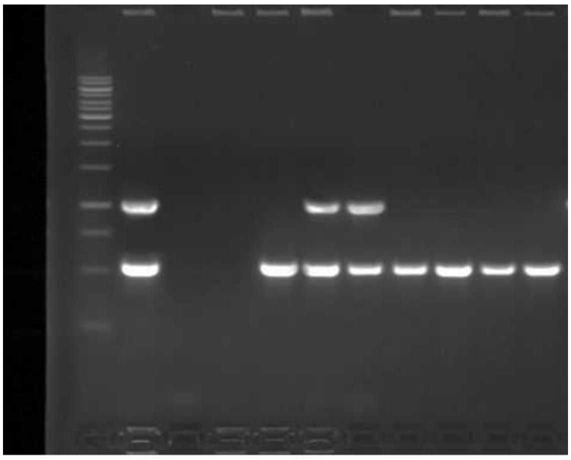

M 1 2 3 4 5 6

**Fig. 3C**

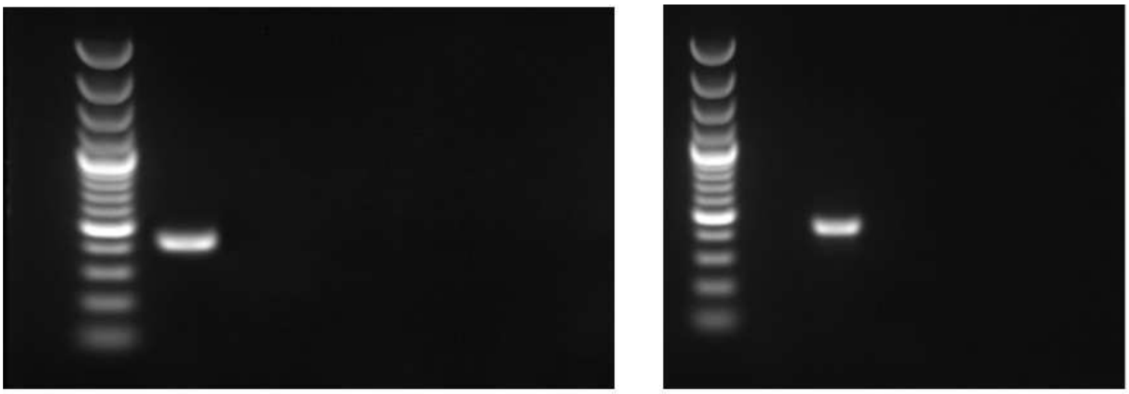

M 1 2 3 4 5 6 M 7

## References


1. Slightom, J.L., Durand-Tardif, M., Jouanin, L. and Tepfer, D. Nucleotide sequence analysis of TL-DNA of *Agrobacterium rhizogenes* agropine type plasmid. *J Biol Chem* **261**:108-121. (1986)
2. Martínez-Márquez A, et al. Production of highly bioactive resveratrol analogues pterostilbene and piceatannol in metabolically engineered grapevine cell cultures. *Plant Biotechnol J* **14**(9):1813-25. (2016)
3. Hirayama, T. et al. Organization and characterization of the virCD genes from *Agrobacterium rhizogenes*. *Molecular and General Genetics*, 213:229-237. (1988)
4. Karimi M, Inzé D, Depicker A. Gateway vectors for Agrobacterium-mediated plant transformation. *Trends Plant Sci*. **7**(5):193-195.
